# Risk of Second Primary Female Genital Malignancies in Women with Breast Cancer: a SEER Analysis

**DOI:** 10.1007/s12672-018-0330-0

**Published:** 2018-03-19

**Authors:** Zhiyu Li, Qi Wu, Junlong Song, Yimin Zhang, Shan Zhu, Shengrong Sun

**Affiliations:** Department of Breast and Thyroid Surgery, Renmin Hospital of Wuhan University, 238 Ziyang Road, Wuhan, 430060 Hubei Province People’s Republic of China

**Keywords:** Breast cancer, Second primary female genital cancer, SEER, Risk factor

## Abstract

Breast cancer survivors are at an increased risk of second primary cancers, and the risk factors for the latter may have clinical significance. The aims of our study were to evaluate the incidences and risk factors of second primary female genital cancers (corpus uteri, cervix uteri plus ovary) in a large cohort of breast cancer survivors. Using the Surveillance, Epidemiology, and End Results (SEER) database, we examined the standardized incidence ratio (SIR) and risk factors for second primary female genital cancers observed between 2000 and 2014. Breast cancer survivors had increased SIRs for second corpus uteri cancers and second ovarian cancers and a decreased SIR for second cervical cancers (SIR 1.17, 1.12, and 0.64, respectively). Risk factors of second corpus uteri cancers were the age at first cancer diagnosis, race (black vs. white, aHR = 1.142 95% CI 1.005–1.298), and progesterone receptor (PR) status (PR+ vs. PR−, aHR = 1.131 95% CI 1.004–1.273). In addition, the risk of second ovarian cancer was positively associated with age while inversely associated with race (black vs. white, aHR = 0.691 95% CI 0.555–0.859) and estrogen receptor (ER) status (ER+ vs. ER−, aHR = 0.655 95% CI 0.544–0.788). Age, race, and hormone receptor status are risk factors of developing second female genital cancers among breast cancer survivors. Older age, black race, and a PR+ status in survivors are associated with a higher risk of second corpus uteri cancers. Additionally, older age and an ER− status should increase vigilance for potential second ovarian cancers.

**Electronic supplementary material:**

The online version of this article (10.1007/s12672-018-0330-0) contains supplementary material, which is available to authorized users.

## Introduction

According to statistics regarding cancer incidence and mortality, breast cancer is the most common malignancy diagnosed in females, accounting for nearly one in three cancers [1]. Breast cancer is also the second leading cause of cancer-related death after lung cancer among women [2]. With the development of the early screening, detection, and systemic treatment of breast cancer, significant improvements in breast cancer survival outcomes have been made. Thus, women with breast cancer can achieve prolonged survival times and better life expectancies. However, a higher risk of developing a second primary female genital cancer among breast cancer survivors may translate into an important health problem in their lifetimes [3]. Within 10 years after the initial diagnosis, approximately 10% of breast cancer patients develop a subsequent primary cancer, including second primary endometrial cancer and second primary ovarian cancer [4].

Previous studies have suggested a consensus in the increased risk of second primary female genital cancers among breast cancer survivors. A large cohort study reported that women with breast cancer had a 30% excess risk for second primary cancers, particularly for endometrial cancer and ovarian cancer [5]. The problem of second primary female genital cancers among breast cancer survivors may be related to treatment side effects or to etiological associations for multiple cancers. With the growing interest in identifying possible relationships between second female genital malignancies and first cancers, studies have focused on multiple factors, such as lifestyle, environment, treatment side effects, and hormonal and/or genetic factors. A European study reported that risk factors of second female genital malignancies included the body mass index and smoking status of breast cancer patients [5]. Moreover, estrogen receptor (ER)-positive breast cancer patients treated with tamoxifen have an increased risk of subsequent endometrial cancer [6, 7]. However, one study has shown that patients with breast cancer have a higher risk of subsequent endometrial cancer regardless of ER or progesterone receptor (PR) status [8].

The aims of our study were to systemically evaluate the incidence of second primary female genital cancers in a large cohort of breast cancer patients and to identify risk factors for second primary corpus uteri cancer and ovarian cancer. Using the Surveillance, Epidemiology, and End Results (SEER) database, we calculated the standardized incidence ratio (SIR) for second primary cancers after breast cancer diagnosed between 2000 and 2014. Meanwhile, we used multivariable analysis to examine the risk factors for the development of a second primary female genital cancer after breast cancer.

## Materials and Methods

### Data Source and Study Design

The SEER program of the National Cancer Institute collects information on cancer incidence, patient survival, and patient characteristics from several geographically defined regions in the USA. Data were selected from the SEER 18 registry database from January 1, 2000, to December 31, 2014. The registries included San Francisco-Oakland SMSA, Connecticut, Detroit (Metropolitan), Hawaii, Iowa, New Mexico, Seattle (Puget Sound), Utah, Atlanta (Puget Sound), San Jose-Monterey, Los Angeles, Rural Georgia, California excluding SF/SJM/LA, Kentucky, Louisiana, New Jersey, and Greater Georgia. The International Classification of Diseases for Oncology, 3rd edition (ICD-O-3) histopathology codes were used to analyze the cases of malignancy.

From this database, we collected data for women with breast cancer only and breast cancer survivors with multiple cancers who were diagnosed between 2000 and 2014. Second primary cancers that were diagnosed within 6 months of breast cancer diagnosis were excluded as these were likely to be pre-existing or synchronous cancers [8]. Cases derived only from death certificates or autopsy were also excluded. Follow-up continued until the date of diagnosis of any second cancer, death from any cause, the date of last known vital status, or the end of the study (December 31, 2014). In the Cox proportional hazards regression model analysis of risk factors for second female genital cancers after breast cancer, none of the follow-up data were excluded.

There were 615,581 women with breast cancer only and 50,681 breast cancer survivors with multiple cancers in this database. A total of 4740 patients developed a second female genital cancer after a primary breast cancer diagnosis, including 3033 women with second corpus uteri cancer, 295 women with cervical cancer, and 1412 with ovarian cancer. Additionally, analyses were conducted based on the characteristics of the first breast cancer, which included the year of breast cancer diagnosis (2000–2004, 2005–2009, and 2010–2014), age at breast cancer diagnosis (age under 40, 40–49, 50–59, 60–69, and 70+ years), latency period (6–11, 12–59, 60–119, and 120+ months), race (white, black, American Indian/Alaskan Native, Asian, or Pacific islander), subtype (ER+PR+, ER+PR−, ER−PR+, ER−PR−), and menopausal status (premenopausal, perimenopausal, and postmenopausal) [9].

### Statistical Analysis

#### Estimation of Standardized Incidence Ratio

To compare the relative risk with the general population, we used the SEER*Stat Multiple primary-standardized incidence ratios (MP-SIR) tool (version 8.3.4) to calculate the SIRs by dividing the observed numbers of second primary cancers by the expected numbers of second primary cancers based on the rates of the general population, along with the 95% confidence interval (95% CI). CIs and *P* values were at 0.05 significance alpha levels and were two-sided based on Poisson exact methods. To avoid statistically unstable estimates, the SIRs and CIs of the 0–20 age group were not presented where the number of observed cases was zero [10].

#### Risk Factors Analysis

The characteristics of the women with breast cancer only and those with second primary female genital cancer were compared with chi-square tests. We used crude semi-parametric Cox proportional hazards regressions to evaluate the hazards ratios (HRs) and corresponding 95% CIs and to show the risk factors for the development of a second primary female genital cancer after breast cancer. The latency period was started at the first breast cancer diagnosis and censored at the second cancer diagnosis. All statistical analyses and charts were generated using SPSS 19.0 (IBM Corporation, Armonk, NY, USA) and GraphPad Prism 6.07. Significance levels were set at *p* value < 0.05. All tests were two-sided.

## Results

### SIRs for Second Female Genital Cancers Among Breast Cancer Survivors

Table 1 shows the SIRs for second primary cervical cancer and second primary corpus uteri cancer among breast cancer survivors. There were 295 cervical cancers and 3033 endometrial cancers after first primary breast cancer (Supplementary Table 1). The SIR for cervical cancer was decreased with statistical significance for patients with first breast cancer, while the SIR for corpus uteri cancer increased for patients with first breast cancer (SIR, 0.64; 95% CI, 0.57–0.72; SIR, 1.17; 95% CI, 1.13–1.21, respectively). Decreased SIRs for cervical cancer patients were observed for all years of breast cancer diagnosis, all ages at breast cancer diagnosis, all latency periods, and all menopausal statuses after breast cancer diagnosis. The SIRs for the ER+ and ER−PR− subtypes were both decreased in patients with cervical cancer. In addition, the increased SIRs for corpus uteri cancer patients were observed in all years of breast cancer diagnosis but declined for more recent time periods (2000–2004 SIR, 1.37; 2005–2009 SIR, 1.26; 2010–2014 SIR, 1.08). The SIRs for all latency periods except the first 6–11 months after breast cancer diagnosis were elevated and increased with the time period (12–59 SIR, 1.13; 60–119 SIR, 1.22; 120+ SIR, 1.27). The SIRs for ER+PR+ and ER− breast cancer were all elevated in patients with corpus uteri cancer.

**Table 1:** Standardized incidence ratios for second cervical cancer and corpus uteri cancer risk in breast cancer patients by characteristic

Characteristics	Cervix uteri		Corpus uteri	
	*O*	SIR (95% CI)	*O*	SIR (95% CI)
Calendar year of breast cancer diagnosis				
2000–2004	33	0.55* (0.38, 0.78)	352	1.37* (1.23, 1.52)
2005–2009	114	0.69* (0.57, 0.83)	1073	1.26* (1.19, 1.34)
2010–2014	148	0.63* (0.54, 0.74)	1608	1.08* (1.03, 1.13)
Age at breast cancer diagnosis, years				
Age under 40	4	0.33* (0.09, 0.84)	9	1.31 (0.60, 2.48)
40–49	36	0.53* (0.37, 0.73)	150	1.55* (1.32, 1.82)
50–59	79	0.67* (0.53, 0.83)	677	1.21* (1.12, 1.30)
60–69	66	0.53* (0.41, 0.68)	916	0.97 (0.91, 1.03)
70+	110	0.81* (0.67, 0.98)	1281	1.30* (1.23, 1.37)
Latency period, months				
6–11	27	0.66* (0.44, 0.97)	202	1.02 (0.89, 1.17)
12–59	140	0.59* (0.49, 0.69)	1423	1.13* (1.07, 1.19)
60–119	106	0.75* (0.61, 0.90)	1074	1.22* (1.15, 1.30)
120+	22	0.60* (0.38, 0.91)	334	1.27* (1.14, 1.42)
Race				
White	228	0.64* (0.56, 0.72)	2507	1.12* (1.08, 1.16)
Black	43	0.69* (0.50, 0.93)	288	1.35* (1.20, 1.51)
American Indian/Alaskan Native	1	0.56 (0.01, 3.12)	7	1.05 (0.42, 2.17)
Asian or Pacific Islander	22	0.64* (0.40, 0.98)	226	1.82* (1.59,2.08)
Menopausal status^1^				
Premenopausal (≤ 45 years)	24	0.54* (0.35, 0.81)	52	1.27 (0.95, 1.67)
Perimenopausal (46–55 years)	60	0.57* (0.43, 0.73)	452	1.37* (1.24, 1.50)
Postmenopausal (56+ years)	211	0.68* (0.59, 0.78)	2529	1.14* (1.09, 1.18)
Subtype				
ER+PR+	169	0.63* (0.64, 0.73)	1818	1.17* (1.11, 1.22)
ER+PR−	25	0.52* (0.34, 0.77)	305	1.04 (0.93, 1.17)
ER−PR+	4	0.71 (0.19, 1.82)	38	1.43* (1.01, 1.96)
ER−PR−	48	0.62* (0.46, 0.83)	439	1.13* (1.03, 1.24)

The bold values indicate the SIR (risk) for developing a second primary cancer was significantly increased

The italic values indicate the SIR (risk) for developing a second primary cancer was significantly decreased

*O* observed numbers, *SIR* standardized incidence ratio, *HR* hormone receptor, *ER* estrogen receptor, *PR* progesterone receptor

*P < 0.05; confidence intervals are 95%

^1^Women were considered perimenopausal if their age is in between 46 and 55 years, and the menopausal status is unknown

Table 2 shows the SIRs for patients with second primary ovarian cancer after first breast cancer (SIR, 1.12; 95% CI, 1.06–1.18). A total of 1412-s primary ovarian cancers were examined and analyzed in our study. The elevated SIRs were observed in groups containing patients under 60 years of age, and the SIRs decreased with increasing age at breast cancer diagnosis (age under 40 SIR, 6.00; 40–49 SIR, 2.43; 50–59 SIR, 1.46). In the 70+ age group, patients with first breast cancer had a decreased risk of developing second ovarian cancer compared to the general group (SIR, 0.89; 95% CI, 0.81–0.96). Elevated SIRs were also observed in the 12–119-month latency periods (12–59 SIR, 1.10; 60–119 SIR, 1.16). Additionally, SIRs for second primary ovarian cancer were significantly increased after ER− breast cancer (ER−PR+ SIR, 2.18; ER−PR− SIR,1.81). In contrast, for the ER+PR+ subtype of breast cancer, there was a decreased risk of developing second ovarian cancer (SIR, 0.92; 95% CI, 0.85–0.99). Meanwhile, premenopausal patients showed higher SIRs than those with other menopausal statuses.

**Table 2:** Standardized incidence ratios for second ovarian cancer risk in breast cancer patients by characteristic

Characteristics	*O*	SIR (95% CI)
Calendar year of breast cancer diagnosis		
2000–2004	195	1.29* (1.12–1.49)
2005–2009	528	1.17* (1.07–1.27)
2010–2014	689	1.04 (0.97–1.13)
Age at breast cancer diagnosis, years		
Age under 40	24	6.00* (3.84–8.93)
40–49	148	2.43* (2.06–2.86)
50–59	325	1.46* (1.31–1.63)
60–69	364	1.03 (0.93–1.14)
70+	551	0.89* (0.81–0.96)
Latency period, months		
6–11	113	1.14 (0.94–1.36)
12–59	686	1.10* (1.02–1.19)
60–119	485	1.16* (1.06–1.27)
120+	128	1.07 (0.89–1.27)
Race		
White	1213	1.09* (1.03, 1.15)
Black	99	1.18 (0.96, 1.43)
American Indian/Alaskan Native	5	1.67 (0.54, 3.90)
Asian or Pacific Islander	95	1.66* (1.35, 2.03)
Menopausal status^1^		
Premenopausal (≤ 45 years)	97	3.67* (2.97, 4.47)
Perimenopausal (46–55 years)	256	1.63* (1.44, 1.84)
Postmenopausal (56+ years)	1059	0.98 (0.92, 1.04)
Subtype		
ER+PR+	695	0.92* (*0.85*–*0.99*)
ER+PR−	151	1.06 (0.89–1.24)
ER−PR+	27	2.18* (1.43–3.17)
ER−PR−	321	1.81*(1.62–2.02)

The bold values indicate the SIR (risk) for developing a second primary cancer was significantly increased

The italic values indicate the SIR (risk) for developing a second primary cancer was significantly decreased

*O* observed numbers, *SIR* standardized incidence ratio, *HR* hormone receptor, *ER* estrogen receptor, *PR* progesterone receptor

*P < 0.05; confidence intervals are 95%

^1^Women were considered perimenopausal if their age is in between 46 and 55 years, and the menopausal status is unknown

### Risk Factors of Developing a Second Female Genital Cancer After Breast Cancer

The clinical characteristics of the women who developed breast cancer only and those who developed second primary female genital cancer are shown in the supplementary Tables. Women with breast cancer only differed from those who developed second primary corpus uteri cancer in terms of the year of breast cancer diagnosis, age at breast cancer diagnosis, race, and ER and PR status, based on significant differences in chi-square tests (Supplementary Table 1). Women with breast cancer only also differed significantly from those who developed second primary ovarian cancer in terms of the year of breast cancer diagnosis, race, and ER and PR status (Supplementary Table 2). With regard to age at breast cancer diagnosis, we found no statistically significant differences between women with breast cancer only and women with second primary ovarian cancer.

**Fig. 1:**
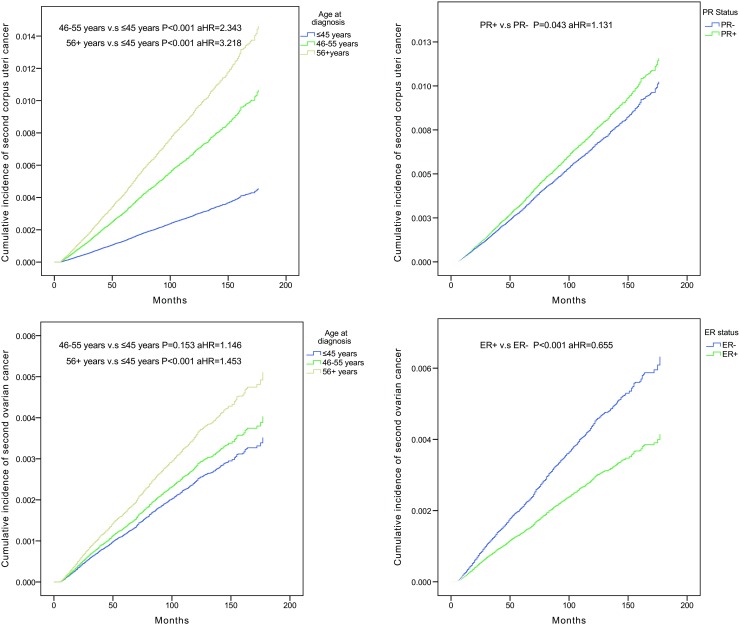
Cumulative incidence curves of second corpus uteri cancer among breast cancer survivors in different age groups. Cumulative incidence curves of second corpus uteri cancer in PR+ breast cancer survivors compared with PR− survivors. Cumulative incidence curves of second ovarian cancer among breast cancer survivors in different age groups. Cumulative incidence curves of second ovarian cancer in ER+ breast cancer survivors compared with ER− survivors

When we considered risk factors for second primary corpus uteri cancer after breast cancer (Table 3), the risk of second primary corpus uteri cancer was positively associated with the age at first cancer (46–55 vs. ≤ 45 years, aHR = 2.343, *P* < 0.001; 56+ vs. ≤ 45 years, aHR = 3.218, *P* < 0.001) (Fig. 1a), race (black vs. white, aHR = 1.142, *P* = 0.042), and PR status (positive vs. negative, aHR = 1.131, *P* = 0.043) (Fig. 1b), while an inverse association was found with the year of first cancer, and ER status showed no association with risk (*P* = 0.293). Our study also demonstrated that the risk of second primary ovarian cancer was positively associated with the year of first cancer and age at first cancer (56+ years vs. ≤ 45 years, aHR = 1.453, *P* < 0.001) (Fig. 1c), while an inverse association was found with race (black vs. white, aHR = 0.691, *P* = 0.001) and ER status (positive vs. negative, aHR = 0.655, *P* < 0.001) (Fig. 1d), and PR status showed no association with risk (*P* = 0.060).

**Table 3:** Cox proportional hazards regression model analysis of risk factors for second female genital cancers after breast cancer: adjusted hazard ratio (aHR) and 95% confidence interval (95% CI)

Variables	Corpus uteri		Ovary	
	aHR (95% CI)	*P* value	aHR (95% CI)	*P* value
Calendar year of breast cancer diagnosis				
2000–2004	Reference		Reference	
2005–2009	0.769 (0.704, 0.841)	**< 0.001**	1.321 (1.104, 1.580)	**0.002**
2010–2014	0.711 (0.616, 0.820)	**< 0.001**	1.840 (1.277, 2.652)	**0.001**
Age at diagnosis				
≤ 45 years	Reference		Reference	
46–55 years	2.343 (1.990, 2.758)	**< 0.001**	1.146 (0.951, 1.382)	0.153
56+ years	3.218 (2.764, 3.746)	**< 0.001**	1.453 (1.231, 1.716)	**< 0.001**
Race				
White	Reference		Reference	
Black	1.142 (1.005, 1.298)	**0.042**	0.691 (0.555, 0.859)	0.001
American Indian/Alaskan Native	0.558 (0.266, 1.173)	0.124	0.783 (0.325, 1.886)	0.586
Asian or Pacific Islander	1.072 (0.928, 1.239)	0.346	0.962 (0.776, 1.194)	0.727
ER				
Negative	Reference		Reference	
Positive	0.928 (0.808, 1.066)	0.293	0.655 (0.544, 0.788)	**< 0.001**
PR				
Negative	Reference		Reference	
Positive	1.131 (1.004, 1.273)	**0.043**	0.848 (0.715, 1.007)	0.060

*P values calculated by Log-rank testing; bold if statistically significant, *P* < 0.05

## Discussion

In this study, we found a significantly decreased risk of second cervical cancer in patients with first breast cancer. Viral carcinogenesis is the main risk factor for cervical cancer. Moreover, a previous study showed the induction of aromatase expression in cervical carcinomas and opened the possibility that the use of aromatase inhibitors (AIs) for primary breast cancer may be a potential favorable factor in the development of subsequent cervical cancer [11]. Additionally, the cervix is targeted by estrogen through estrogen receptor alpha and beta (ER-α and ER-β). ER-α expression occurs mainly in the normal cervical epithelium but is lost in cervical cancer. However, ER-β is expressed in both the normal cervix and cervical cancer. One study has suggested that estrogen may affect ER-β, which is a suppressor of ER-α in carcinogenesis [12]. Thus, AI therapy for postmenopausal breast cancer patients and ER-β expression might be the causes of the decreased risk of second cervical cancer among breast cancer survivors.

Our study showed that the SIR for second primary corpus uteri cancer was significantly increased in patients with first breast cancer regardless of ER and PR status. Previous studies have confirmed an increased risk of second corpus uteri cancer after ER-positive breast cancer in patients with tamoxifen therapy compared to the general population [13, 14]. Interestingly, ER−PR− breast cancer patients who were less likely to receive tamoxifen therapy also had a significantly increased SIR for second corpus uteri cancer, and this result suggests that tamoxifen therapy may not be the only potential reason for second corpus uteri cancer after initial breast cancer. The results of a recent study were consistent with our study, which showed that breast cancer patients with different hormone receptor statuses had increased risks of subsequent corpus uteri cancer [8]. In addition to tamoxifen therapy, shared BRCA1/2 gene mutations may contribute to a higher risk of corpus uteri cancer among breast cancer patients, compared to the general population [15]. Our study suggested a trend in which the SIR of second corpus uteri cancer was declining more in recent years. However, this trend may be merely a reflection of the shorter latency period for breast cancer survivors more recently. On the one hand, Table 1 shows that the risk of second uterine corpus cancer increases with the latency period. On the other hand, a large case-control study found that patients using tamoxifen for less than 2 years failed to show a statistically increased risk of developing uterine corpus cancer [16]. Our study continued until December 31, 2014, and revealed that numerous breast cancer patients diagnosed from 2010 to 2014 may have undergone tamoxifen treatment for less than 2 years, so the effect of tamoxifen on second uterine corpus cancer development among these patients was underestimated.

Our study suggested that survivors had a higher risk of developing second primary ovarian cancer after the first breast cancer than the general population, which was in line with previous studies [17, 18]. The results further revealed that the young breast cancer survivors had a higher risk of second primary ovarian cancer than young women in the general population, and the peak of this risk was particularly notable in the 30–39 age group but lowest in the 70+ age group. This may be associated with a common germline mutation (BRCA1) and similar hormonal exposure. To some extent, BRCA1 mutations expressly cause breast cancer at a young age [19]. In addition, oophorectomy, a treatment for breast cancer, can lead to an incidental finding of early ovarian cancer. The SIRs for second primary ovarian cancer were significantly increased after ER-negative breast cancer. This increase may be related to the common BRCA1 gene for both early-onset ER-negative breast cancer and ovarian cancer [20]. Surprisingly, for both corpus uteri and ovarian sites, women with ER−PR+ breast cancer appeared to have the highest risk. However, progesterone signaling should not be viewed as a risk factor for both uterine corpus and ovarian cancers. Progesterone is a favorable prognostic factor and inhibits cancer cell growth and metastasis development in both uterine corpus cancer and ovarian cancer [21]. ER−PR+ breast cancer is rare because PRs are ER-induced genes and because PR positivity is driven by an active ER. Thus, the expression of ER−PR+ may be induced by other signaling. This alternative signaling may explain how ER−PR+ breast cancers are associated with earlier recurrence times and poorer overall survival rates than ER+PR+ breast cancers [22]. Patients with ER−PR+ breast cancers still receive tamoxifen therapy and are influenced by the side effects of tamoxifen, but these patients have poorer outcomes than ER+PR+ patients.

In contrast to previous studies, we had extensive information on cancer risk factors for second female genital cancers, including the year of breast cancer diagnosis, age at breast cancer diagnosis, race, and hormone receptor status. For breast cancer survivors, in the development of second primary female genital cancer, older age (45+ years), black race, and PR+ breast cancer tended to be associated with a higher risk of second corpus uteri cancer. Meanwhile, older age (56+ years), white race, and ER- breast cancer needed heightened vigilance for potential second ovarian cancer. Elevated risks were noted in patients who were perimenopausal or postmenopausal, which may indicate an age-related preference in the risk of second primary female genital cancer among breast cancer patients. However, ER+ breast cancer patients had a decreased risk of developing second ovarian cancer compared to ER− patients. This decreased risk may be due to hormone therapy for first breast cancer. Nevertheless, the SEER database does not contain complete treatment information to evaluate the impact of hormone therapy.

Our study had strengths and weaknesses. This study was based on a large, well-established, and standardized population database. However, the heterogeneous population and retrospective analysis of the SEER data were the main limitations. In addition, patients with first primary breast cancer tend to be given more thorough attention than the general population, and a surveillance bias exists for patients with second primary cancer. To address these issues, we analyzed data from a 15-year period and data from 18 registries, making these systemic errors non-differential. Potential pre-existing or synchronous cancers were another concern. Therefore, patients with second primary cancers diagnosed within 6 months of breast cancer diagnosis were excluded from our cohort. Moreover, due to the composition of the dataset, the numbers of patients for second female genital cancers in some race and age groups of breast cancer patients were small. These relevant findings would need further confirmation.

In conclusion, this large population-based study suggests that women with first primary breast cancer have higher risks of developing second primary corpus uteri cancer and second primary ovarian cancer than the general population. In contrast, breast cancer survivors have a decreased risk of developing second primary cervical cancer. These increased and decreased risks of developing second primary cancers suggest associations with breast tumor biology and shared genetic susceptibility. Meanwhile, several risk factors were associated with an increase (age at first cancer, race, PR status) or a decrease (year of first cancer) of second primary corpus uteri cancer and with an increase (year of first cancer, age at first cancer) or a decrease (race, ER status) of second primary ovarian cancer. These findings are useful for health planning, including screening and the development of specific guidelines for both the general population and breast cancer patients. Further work is needed to explore possible reasons for this association and the survival outcomes of these patients.

## Electronic supplementary material

Supplementary Table 1 Breast Cancer Patient Characteristics within Subgroups (DOCX 16 kb)

Supplementary Table 2 Breast Cancer Patient Characteristics within Subgroups (DOCX 15 kb)
