# Supplementary material for: Risk of Second Primary Female Genital Malignancies in Women with Breast Cancer: a SEER Analysis
Source: Horm Cancer. 2018 Mar 19;9(3):197–204. doi: 10.1007/s12672-018-0330-0 (PMC5945714; doi:10.1007/s12672-018-0330-0)
Supplement: Supplementary file 2 — Breast Cancer Patient Characteristics within Subgroups (DOCX 15 kb) [file 12672_2018_330_MOESM2_ESM.docx]

**Supplementary Table 2.** Breast Cancer Patient Characteristics within Subgroups

| **Variables** | **Women with breast cancer only**  **N=615,581(%)** | **Women with second ovarian cancer**  **N=1,267(%)** | **P value^*^** |
| --- | --- | --- | --- |
| **Calendar year of breast cancer diagnosis** |  |  | **< 0.001** |
| **2000-2004** | 186,582(30.3) | 758(59.8) |  |
| **2005-2009** | 200,844(32.6) | 382(30.1) |  |
| **2010-2014** | 228,155(37.1) | 127(10.0) |  |
| **Age at diagnosis, y** |  |  | 0.127 |
| **≤45 years** | 95,792(15.6) | 173(13.7) |  |
| **46-55 years** | 149,569(24.3) | 303(23.9) |  |
| **56+ years** | 370,220(60.1) | 791(62.4) |  |
| **Race** |  |  | **<0.001** |
| **White** | 494,911(80.4) | 1,084(85.6) |  |
| **Black** | 65,946(10.7) | 88(6.9) |  |
| **American Indian/Alaska Native** | 3,308(0.5) | 5(0.4) |  |
| **Asian or Pacific Islander** | 47,324(7.7) | 90(7.1) |  |
| **Unknown** | 4,092(0.7) | 0(0.0) |  |
| **ER** |  |  | **< 0.001** |
| **Negative** | 113,977(18.5) | 312(24.6) |  |
| **Positive** | 445,027(72.3) | 789(62.3) |  |
| **Borderline** | 850(0.1) | 1(0.1) |  |
| **Unknown** | 55,727(9.1) | 165(13.0) |  |
| **PR** |  |  | **< 0.001** |
| **Negative** | 174,170(28.3) | 428(33.8) |  |
| **Positive** | 377,352(61.3) | 646(51.0) |  |
| **Borderline** | 2,611(0.4) | 10(0.8) |  |
| **Unknown** | 61,448(10.0) | 183(14.4) |  |

** P values calculated by Pearson Chi squared testing; Bold if statistically significant, P < 0.05*
